# Potentials of community-based early detection of cardiovascular disease risk during the COVID-19 pandemic

**DOI:** 10.1186/s12889-021-11384-6

**Published:** 2021-07-04

**Authors:** Kemal Nazarudin Siregar, Rico Kurniawan, Ryza Jazid BaharuddinNur, Dion Zein Nuridzin, Yolanda Handayani, Retnowati, Rohjayanti, Lindawati Halim

**Affiliations:** 1 Department of Biostatistics and Population Studies, Faculty of Public Health, Universitas Indonesia, Depok, Indonesia; 2 Health Informatics Research Cluster, Faculty of Public Health, Universitas Indonesia, Depok, Indonesia; 3 Research Center of Biostatistics and Health Informatics, Faculty of Public Health, Universitas Indonesia, Depok, Indonesia; 4 Head of Section of NCD Control Program, District Health Office, Bogor District, Indonesia; 5 Coordinator of Health Centers, Babakan Madang Sub-district, Bogor District, Indonesia

**Keywords:** Early detection, Cardiovascular disease, Community-based, COVID-19, Mhealth

## Abstract

**Background:**

The Coronavirus Disease 2019 (COVID-19) pandemic has led to a significant decline in Non Communicable Diseases (NCD) screening and early detection activities, especially Cardiovascular Disease (CVD). This study aims to assess the potential of community-based self-screening of CVD risk through the mhealth application.

**Methods:**

This is operational research by actively involving the community to carry out self-screening through the mHealth application. Community health workers were recruited as facilitators who encourage the community to carry out self-screening. To evaluate the potential of community-based self-screening of CVD risk, we use several indicators: responses rate, level of CVD risk, and community acceptance.

**Results:**

Of the 846 individuals reached by the cadres, 53% or 442 individuals carried out self-screening. Based on the results of self-screening of CVD risk, it is known that around 21.3% are at high risk of developing CVD in the next 10 years. The results of the evaluation of semi-structured questions showed that about 48% of the people had positive impressions, 22% assessed that this self-screening could increase awareness and was informative, 3% suggested improvements to self-screening tools.

**Conclusion:**

Cadres play an important role in reaching and facilitating the community in their environment to remain aware of their health conditions by conducting self-screening of CVD risk. The availability of the mHealth application that the public can easily access can simplify CVD risk prediction and expand screening coverage, especially during the COVID-19 pandemic, where there are social restrictions policies and community activities.

## Introduction

CVD is currently one of the leading causes of death, accounting for about one-third of all deaths worldwide, four-fifths of which occur in developing countries [1]. In Indonesia, the main cause of morbidity and mortality is CVD responsible for one-third of all deaths [2]. Some of the main risk factors for CVD are metabolic factors associated with hypertension, diabetes mellitus, and hypercholesterolemia. In 2018, the Indonesian Basic Health Research showed that the proportion of the most common diseases was hypertension at 63.5%, diabetes mellitus at 5.7%, heart disease at 4.5%, and stroke at 4.4% [3]. The Indonesian Basic Health Research has reported an increasing trend in hypertension, diabetes mellitus, and stroke in Indonesia [4]. Observing the development of the CVD problem in Indonesia, which the COVID-19 pandemic has exacerbated, it is necessary to prepare more responsive public health efforts to control CVD.

The most effective management of CVD is focused on screening and early detection of preventable risk factors. Studies have shown that preventing disease and death from CVD will not be effective without population-based risk factor investigations and strengthening public awareness about CVD prevention [5–7]. The data regarding the distribution of CVD risk factors in the community is currently insufficient to determine the appropriate intervention. Therefore, a community-based early detection mechanism for CVD risk factors needs to be developed to strengthen the disease control program.

Addressing this problem, the Government of Indonesia has actually implemented a community-based control program under the health center at the sub-district level. The program’s main target so far is the elderly. However, this program is underutilized. It is marked by low coverage, and since the COVID-19 outbreak, this program has almost stopped. The COVID-19 pandemic has led to a significant decline in CVD screening and treatment [8]. Thus, the research question is, how can CVD control activities be reactivated so that early detection in the community during the COVID-19 pandemic can be conducted properly?

A community-based participatory program (CPBR) is an approach to involve the community in research actively. Research conducted by Tremblay et al. using the CBPR approach can show significant processes and results such as creating awareness; shifting norms and beliefs about NCD in the community; fostering community mobilization, collaboration, and leadership around this issue; building community capacity, skills, and expertise in NCD prevention; creating a culture of collaboration and resource sharing among community organizations and permeating the NCD prevention agenda into other organizations [9]. CBPR enables researchers to understand better community strengths, challenges, and opportunities [10].

This study aims to assess the potential of community-based self-screening of CVD risk through the mhealth application. We propose this research’s novelty because it provides self-screening mechanisms and tools that are easily accessible to the public, especially during a pandemic and even after the pandemic is over.

## Methods

### Building a community-based participatory program

To obtain the potential for self-screening and early detection of CVD risk in the community, we conducted an operational study in Babakan Madang District, Bogor Regency, in September–November 2020. Babakan Madang District consists of nine villages. We recruited two cadres in each village. Cadre or community health workers, voluntary-based health workers, were prepared to reach out and encourage community members’ active participation in self-screening and early detection of CVD.

The recruited cadres were given training on CVD and the mHealth application as a self-screening and early detection of CVD risk. Furthermore, the cadres are asked to actively reach the community in their environment to encourage the community to carry out independent early detection through the mhealth application. In this study, we asked each cadre to reach at least 25 people aged 15 years and over in their environment who can access an early detection tool for CVD risk using mHealth. In the outreach process, the cadres then broadcast WhatsApp messages that contain written informed consent and the link to access the tool for early detection of CVD risk to the community. People who receive messages from cadres independently conduct the early detection of CVD risk by filling out an online form using the mHealth application.

### Development of mHealth for cardiovascular disease risk assessment

The CVD risk early detection form was modified and adapted according to WHO Stepwise [11]. The CVD risk early detection form is then translated into an online form that can be accessed easily with a smartphone using an open data kit (ODK) platform. The use of the ODK platform as a mHealth application here because is considered quite easy and simple, so it is expected that the public can use it easily.

When the community has completed the early detection independently, they will get the CVD risk calculation results automatically displayed on the online form. Automatically, the mHealth application will send the database of the results of the community self-screening to a server that can be accessed by health workers so that the data becomes a reference for health workers to strengthen the program.

mHealth performed the CVD risk assessment by calculating a risk index based on behavioral indicators using the simple risk score (EZ-CVD) [12]. According to the respondent’s answer, the risk index is calculated automatically in the mHealth application based on the weighted results of the risk factor indicators. The results of this risk index calculation are categorized into high risk (score ≥ 20%) and low risk (score <  20%). The risk index can be read directly by respondents via mHealth. The risk assessment data were then analyzed descriptively to assess mHealth’s ability to detect risk factors for CVD in the community.

### Evaluation of community-based early detection of CVD risk

We use three indicators to evaluate the potential of community-based Early Detection of Cardiovascular Disease Risk, that are response rate, the level of CVD risk, and the community acceptance regarding self-screening mechanism. We assessed the response rate of CVD risk self-screening by comparing people aged 15 years and over who conducted self-screening with the total individual reached by cadres through broadcasts messages. mHealth performed the level of CVD risk by calculating a risk index based on behavioral indicators using the simple risk score (EZ-CVD) [12]. According to the respondent’s answer, the risk index is calculated automatically in the mHealth application based on the weighted results of the risk factor indicators. The results of this risk index calculation are categorized into high risk (score ≥ 20%) and low risk (score <  20%). The risk index can be read directly by respondents via mHealth. The risk assessment data then analyze descriptively to assess mHealth’s ability to detect risk factors for CVD in the community. The acceptance level of self-screening assessed by open-ended questions to the community who conduct self-screening through mHealth.

### Ethical Clearence

This research has received ethical approval by the ethics commission in the Faculty of Public Health Universitas Indonesia register number 538/UN2.F10.D11/PPM.00.02/2020.

## Results

The COVID-19 pandemic has caused many community-based health programs to fail, one of which is the early detection of CVD. Limiting community-based social activities causes low access to health services, especially for promotive and preventive activities. One strategy to overcome this problem is to empower cadres and use appropriate technology.

### Community responses for CVD risk self-screening

**Fig. 1:**
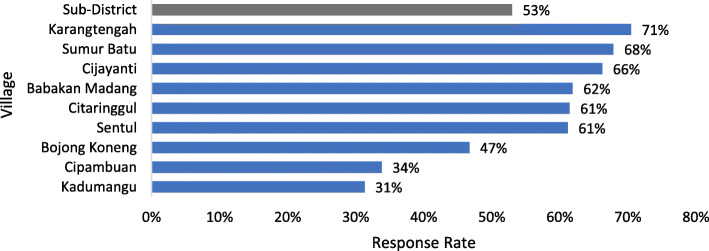
Response Rate of mHealth User in the Bababakan Madang Sub-District

This research has succeeded in making a tool in the form of mHealth to perform the CVD risk early detection. The results illustrate that the mHealth application is suitable for using the community as an early detection tool for CVD during the COVID-19 pandemic. Out of the 829 respondents contacted by the cadres through WhatsApp messages, their response rate to using the mHealth application was relatively high, with an average of 53% (442 respondents) (see Fig. 1). Out of the nine villages, six had high response rates, ranging from 61 to 71%. Only three villages had low response rates below 50%.

The reasons for those who did not use the mHealth application, among others, admitted that it was not easy to use, they could not access the Internet, or they did not understand the contents of the self-assessment form questions.

### Cardiovascular disease risk assessment and health promotion

This research has adopted a CVD risk assessment that has been compiled into mHealth, where users can automatically get health promotions according to the level of risk they have. This study found that mHealth has been able to show respondents’ risk predictions for suffering from CVD in the next 10 years. Overall, mHealth can detect that around 78.7% of respondents are at low risk (with a score of < 20%) and approximately 21.3% of respondents are at high risk (with a score of ≥20%) of experiencing CVD (Table 1). The indicators used to predict the CVD risk indexes are age and gender. Based on calculating the risk index using the mHealth application, it is known that the average age of respondents who have a high risk is 36 years (age range 25 to 44 years). Men have a higher risk of developing CVD than women.

**Table 1:** Characteristics of Respondents Using the mHealth Application Based on the CVD Risk Index in the Babakan Madang Sub-District

Characteristics of Respondent	Overall (*n* = 442)	Predicted 10-years CVD Risk	
		Low Risk (< 20%) (*n* = 348)	High Risk (≥ 20%) (*n* = 94)
Age, mean (IQR)	32 (24–40)	31 (23–38)	38 (25–44)
Age (years), n (%)*			
15–24	120 (27.1)	100 (83.3)	20 (16.7)
25–34	132 (29.9)	112 (84.8)	20 (15.2)
35–44	133 (30.1)	101 (75.9)	32 (24.1)
45–54	45 (10.2)	29 (64.4)	16 (35.6)
55–63	12 (2.7)	6 (50.0)	6 (50.0)
Sex, n (%)*MaleFemale	112 (25.3)330 (74.7)	39 (34.8)309 (93.6)	73 (65.2)21 (6.4)
Level of Education, n (%)			
Never Attended School	5 (1.1)	4 (80)	1 (20)
Not-completed in Primary	15 (3.4)	14 (93.3)	1 (6.7)
Primary School	88 (19.9)	72(81.8)	16 (18.2)
Junior High School	105 (23.8)	87 (82.9)	18 (17.1)
Senior High School	184 (41.6)	138 (75.0)	46 (25)
Tertiary (University)	45 (10.2)	33 (73.3)	12 (26.7)
Profession, n (%)			
Unemployed	184 (41.6)	169 (91.8)	15 (8.2)
Student	31 (7.0)	28 (90.3)	3 (9.7)
Civil Servant	9 (2)	5 (55.6)	4 (44.4)
Farmer	4 (0.9)	2 (50)	2 (50)
Laborer	66 (14.9)	36 (54.5)	30 (45.5)
Entrepreneur	58 (13.1)	37(63.8)	21 (36.2)
Others	90 (20.3)	71 (78.9)	19 (21.1)

* CVD Risk Index Calculation Indicator

The mHealth application is equipped with a feature that can calculate a risk index. The indicators used to calculate the risk index are smoking behavior, alcohol consumption, history of diabetes, history of hypertension, and family history of premature myocardial infarction. Based on the results of the risk index assessment, it is known that a small proportion of respondents are smokers, namely, 71 respondents (16.1%), and 90% of them are high risk (Table 2). All respondents who have a history of diabetes mellitus are predicted to have a high risk of suffering from CVD in the next 10 years.

**Table 2:** Distribution of Respondents’ Risk Factors Using the mHealth Application Based on the CVD Risk Index in the Babakan Madang Sub-District

Risk Factors, n (%)	Overall (n = 442)	Predicted 10-years CVD Risk	
		Low Risk (< 20%) (n = 348)	High Risk (≥ 20%) (n = 94)
Smoking*			
Never	335 (75.8)	331 (93.4)	22 (6.6)
Former	36 (8.1)	28 (77.8)	8 (22.2)
Current	71 (16.1)	7 (9.9)	64 (90.1)
Alcohol			
Never	419 (94.8)	343 (81.9)	76 (18.1)
Former	12 (2.7)	4 (33.3)	8 (66.7)
Current	11 (2.5)	1 (9.1%)	10 (90.9)
Physical Activity			
Seldom/Rare	61 (13.8)	119 (77.8)	34 (22.2)
Sometimes	228 (51.6)	177 (77.6)	51 (22.4)
Often/Very often	153 (34.6)	52 (85.2)	9 (14.8)
Dietary Fruits and			
Vegetables			
1–2 Servings/day	300 (67.9)	235 (78.3)	65 (21.7)
>2–3 Servings/day	142 (32.1)	113 (79.6)	29 (20.4)
History of Diabetes			
Mellitus*			
Yes	21 (4.8)	0 (0)	21 (100)
No	421 (95.2)	348 (82.7)	73 (17.3)
History of Hypertension*			
Yes	62 (14.0)	39 (62.9)	23 (37.1)
No	380 (86.0)	309 (81.3)	71 (18.7)
Family History of Premature			
Myocardial infarction (MI)*			
Yes	34 (7.7)	17 (50)	17 (50)
No	408 (92.3)	331 (81.1)	77 (18.9)

* CVD Risk Index Calculation Indicator

### User response and accepatance of the community-based early detection of CVD risk

Of the 82 users of the mHealth application, 47 respondents (70%) gave positive responses. As many as 18 respondents (22%) reported that the mHealth application was useful and informative, 39 (48%) showed positive appreciation for the application. Besides functioning as early detection, mHealth can also provide health promotion messages regarding CVD prevention. In this regard, respondents acknowledged that the mHealth application was useful, informative, and well-accepted (Table 3).

**Table 3:** Community Responses

Selected Themes (*N* = 82)	n	%	Representative Quotations
Positive Sentiment			
Increased awareness and informativ	18	22%	*“By filling in this form, I have more knowledge.”* *“Very useful.”* *“Very good and helps for a healthy lifestyle.”* *“I want to live a healthy life.”*
Positive impression	39	48%	*“I like this online mechanism.”* *“Very good.”* *“Very satisfied.”*
Barriers			
Hard to understand the questions	6	7%	*“The question is difficult to understand.”* *“The question needs to be simplified.”*
Too much and wordy questions	15	18%	*“Too many questions.”* *“The question is too wordy.”*
Suggestions			
Not agree with online mechanism	1	1%	*“Actually, through this online filling, (I) don’t agree because some don’t have a cellphone.”*
Improve the tools	3	3%	*“There are more questions about the symptoms experienced because hypertension is usually the initial symptom with dizziness, so it can be distinguished that a headache is a symptom of hypertension or just a normal headache.”*

Of the total participants who gave responses, 25% gave critical responses indicating that the questions were difficult to understand (7%) or too numerous and wordy (18%). The rest, a small proportion of respondents (4%), gave suggestions for improvements to the mHealth application in the future.

## Discussion

### The extent to which early detection of CVD can be applied in the COVID-19 pandemic situation

To date, the NCD control program at the health center is a public service aimed at the elderly and has not been able to reach the productive age group, considering that this group is still active and has high mobility [8, 12]. Research conducted by Subhah et al. (2019) showed that the participation of the productive age population (15–45 years) in NCD prevention was only 45.38% [13]. However, prevention at an early age, namely in young adults, will be more efficient than the cost of treatment for the elderly [14].

The use of mHealth application, supported by community-based participatory programs, is proven to reach a high coverage (87.1%) of the productive age population. Through previous research, we can further develop this study because of the support system that has been established in the form of a community-based health information system, which is supported by community empowerment and the use of mHealth [15]. Furthermore, community empowerment appears to be an essential factor in the successful use of mHealth for early detection [16–19].

The COVID-19 pandemic condition, which limits mobility, has motivated this research to build online communication via WhatsApp, starting from researchers, cadres to community members, who are then encouraged to carry out self-assessments using mHealth. Henry et al.’s [20] research stated that WhatsApp is an innovation that can support community-based communication during an emergency outbreak.

### The extent to which the features in the mHealth application can perform early detection of CVD

**Fig. 2:**
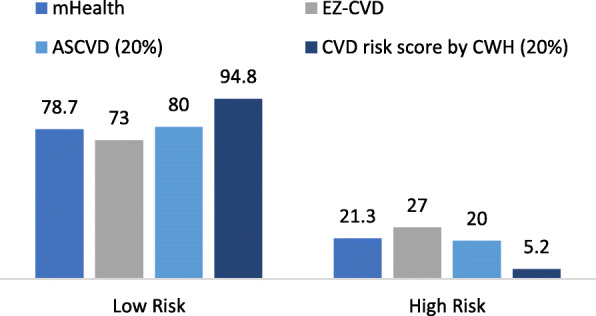
CVD Risk Score

The risk index analysis in the mHealth application refers to the EZ-CVD risk score, which consists of six risk predictor items. It has several advantages compared to the recommended guidelines for the risk score for Atherosclerotic Cardiovascular Disease (ASCVD) [12]. By referring to the EZ-CVD risk score, this mHealth application develops a self-assessment and does not require laboratory testing as early detection to predict CVD risk. Furthermore, Mansoor et al. (2019) argued that the inability to access comprehensive examinations is a major limitation that can result in many patients being missed for CVD risk assessment and receiving recommendations for preventive therapy. A similar study conducted at LMIC showed a slightly higher risk prediction value using a Community Health Workers-based (CHW-based) model for CVD screening (see Fig. 2) [21]. The results of the analysis of the risk score on the mHealth application meanwhile show a proportion of CVD risk that is more or less comparable to the results of EZ-CVD and ASCVD (see Fig. 2) [10, 20]. Even mHealth that is not equipped with laboratory test results has a proportional rate close to ASCVD, which has a high sensitivity for identifying future CVD events of around 80%, with a fairly high specificity (69%) and a positive predictive value (17%) [22]. These findings indicate that at least the mHealth application easily provides CVD risk predictions. In the future, of course, it is necessary to undertake further research to show the extent of the sensitivity and specificity of this application as a tool for the early detection of CVD.

### The extent to which the mHealth application can serve as a source for health promotion

This study obtained feedback from 82 respondents. We found that 70% of them acknowledged that the mHealth application was useful in providing health promotion suitable for their individual needs. The mHealth applications can be classified into two categories: applications designed for disease management and applications that can support user-health behavior changes [23]. Currently, the mHealth application is increasingly being used to promote changes in user behavior to prevent NCDs [24]. Besides being designed to detect CVD risk, the mHealth application is also designed to provide information on health conditions and follow-up recommendations for CVD prevention and control for its users. Research conducted by Handayani et al. [25] showed the factors that determine the successful use of the mHealth application in Indonesia, one of which is the availability of relevant information according to user needs. It seems that the features in the mHealth application are proven to be acceptable to users, especially its ability to provide personal information that is given directly to each user.

### Limitations

The study population was the community of the Babakan Madang sub-district, who were selected purposively by cadres. Hence, the results of this study could not be generalized to other community groups. Furthermore, we did not include several potential CVD risk factors requiring physical and laboratory examinations in this study. We rely on self-assessment results for behavioral risk factors reported by respondents via the mHealth application. This can lead to misclassification of the diagnosis in some individuals who may have an undiagnosed condition. However, this mHealth application is used as an early detection method to identify individuals who require preventive therapy and follow-up recommendations according to the CVD risk calculation in the next 10 years.

## Conclusions

The implementation of mHealth supported by community-based participatory programs can reach the productive age group of 15–45 years, compared to the existing CVD control program that can only reach the elderly group (over 45 years). The mHealth application developed in this study is equipped with a CVD risk calculation that can make predictions with a proportion of the risk index comparable to ASCVD, which has high sensitivity and specificity compared to other detection tools. In fact, the mHealth application is proven to simplify CVD risk assessment without using a laboratory so that people can undertake risk self-assessments even though they have to stay at home because of the COVID-19 outbreak. In addition, mHealth is equipped with a health promotion feature of providing information and follow-up recommendations for users according to their CVD risk index.

## Data Availability

The datasets used and/or analysed during the current study are available from the corresponding author on reasonable request.

## Abbreviations

ASCVD: Atherosclerotic Cardiovascular Disease
CHW: Community Health Worker
COVID-19: Coronavirus Disease 2019
CVD: Cardiovascular Disease
EZ-CVD: A Simple Risk Score for CVD
mHealth: Mobile Health
MI: Myocardial infarction
NCD: Non-Communicable Disease
WAG: WhatsApp Group
